# Multisectoral Emergence of Multidrug-Resistant *Campylobacter coli* Sequence Type 10042 Lineage, Europe, 2018–2025

**DOI:** 10.3201/eid3210.260512

**Published:** 2026-10

**Authors:** Mónica Azevedo, Alexandra Nunes, Maria-Leonor Lemos, Paulo Martins Costa, Ana Amaro, Lurdes Clemente, Célia Leão, Sónia Ramos, Isabel Santos, Katrine Grimstrup Joensen, Catherine Ragimbeau, Lena de Baets, Joël Mossong, Sangeeta Banerji, Antje Flieger, Diana Espadinha, William Byrne, Oda van den Berg, Andries Augustus Kampfraath, Quentin Jehanne, Philippe Lehours, João Paulo Gomes, Mónica Oleastro

**Affiliations:** National Institute of Health Doutor Ricardo Jorge (INSA), Lisbon, Portugal (M. Azevedo, A. Nunes, M.-L. Lemos, J.P. Gomes, M. Oleastro); Research in Veterinary Medicine (I-MVET)/Animal and Veterinary Research Center (CECAV), Faculty of Veterinary Medicine, Lusófona University—Lisbon University Centre, Lisbon (A. Nunes, S. Ramos, I. Santos, J.P. Gomes); ICBAS-Institute of Biomedical Sciences Abel Salazar, University of Porto, Porto, Portugal (M.-L. Lemos, P.M. Costa); Laboratory of Bacteriology and Mycology, National Institute of Agrarian and Veterinary Research, Oeiras, Portugal (A. Amaro, L. Clemente, C. Leão); Statens Serum Institut, Copenhagen, Denmark (K.G. Joensen); Laboratoire National de Santé, Dudelange, Luxembourg (C. Ragimbeau); Laboratoire Vétérinaire et Alimentaire, Division of the Luxembourg Veterinary and Food Administration, Dudelange (L. de Baets); Ministère de la Santé et de la Sécurité Sociale, Direction de la Santé, Luxembourg, Luxembourg (J. Mossong); Robert Koch-Institute, Wernigerode, Germany (S. Banerji, A. Flieger); Public Health Laboratory Dublin, Health Service Executive, Dublin, Ireland (D. Espadinha); Backweston Complex, Celbridge, Ireland (W. Byrne); National Institute of Public Health and the Environment (RIVM), Bilthoven, the Netherlands (O. van den Berg); European Centre for Disease Prevention and Control, Stockholm, Sweden (O. van den Berg); Wageningen Bioveterinary Research, Wageningen UR, Lelystad, the Netherlands (A.A. Kampfraath); French National Reference Centre for Campylobacters and Helicobacters, Bordeaux Hospital University Centre, Bordeaux, France (Q. Jehanne, P. Lehours); University of Bordeaux, INSERM, Bordeaux Institute of Oncology, Bordeaux (P. Lehours)

**Keywords:** *Campylobacter coli* ST10042, antimicrobial resistance, bacteria, whole-genome sequencing, food-borne infections, multidrug resistance, cross-border transmission, One Health, Europe

## Abstract

*Campylobacter* spp. remain the leading bacterial cause of foodborne gastroenteritis in Western countries. We report sustained, previously unrecognized circulation of a multidrug-resistant *Campylobacter coli* sequence type 10042 lineage across Europe during 2018–2025. Whole-genome sequencing of 217 isolates from 9 countries spanning human, animal, food, and environmental sources identified cross-border clusters, including a large lineage linking primarily Portugal and Luxembourg, as well as Germany, Ireland, Spain, and the United Kingdom. Genomic data indicates ongoing clonal expansion with increasing diversification over time. Isolates exhibited a conserved multidrug-resistant phenotype, including resistance to fluoroquinolones, tetracyclines, and β-lactams; reduced susceptibility to carbapenems was observed. Resistance was associated with GyrA Thr86Ile, *tet*(O/32/O)/*tet*(O) genes, and *bla*_OXA-61_ promoter variants; variation in *porA* was linked to variable amoxicillin/clavulanic acid and ertapenem susceptibility. Those findings demonstrate that *C. coli* infections can involve sustained international transmission rather than sporadic cases and highlight the need for coordinated, cross-sector genomic surveillance to detect emerging antimicrobial-resistant lineages.

Since 2005, campylobacteriosis has been the most frequently reported foodborne zoonosis in the European Union (EU); 168,396 human cases were reported in 2024 (*1*). Infections affect all age groups but are most severe in young children, the elderly, and immunocompromised persons (*2*). Campylobacteriosis typically manifests as bloody or watery diarrhea, abdominal cramping, fever, vomiting, and nausea (*3*,*4*). Severe or prolonged infections can lead to complications, such as bacteremia, that occasionally require hospitalization or result in death (*3*,*4*). Antimicrobial therapy is warranted in those cases, highlighting growing concerns regarding *Campylobacter* antimicrobial resistance (AMR), particularly to fluoroquinolones, macrolides, and carbapenems (*4*,*5*).

Campylobacteriosis cases are primarily caused by *Campylobacter jejuni* (≈90%) and *C. coli* (≈10%) (*2*). Poultry is the main reservoir, although livestock commodities, domestic and wild animals, humans, and contaminated water or soil also contribute to transmission (*2*,*6*). Although most *Campylobacter* infections have historically been considered sporadic (*7*), recent whole-genome sequencing (WGS)–based surveillance has demonstrated that many cases occur as genetically related clusters (*8*–*12*).

Genomic surveillance leveraging WGS-based approaches, including whole-genome and core-genome multilocus sequence typing (MLST), has markedly enhanced identification of outbreaks, investigation of transmission pathways, and detection of emerging epidemic lineages, providing high-resolution insights into genetic diversity, AMR determinants, and source attribution (*8*–*12*). WGS data also expand knowledge on novel AMR determinants, such as *porA* gene, which encodes the major *Campylobacter* outer-membrane porin and whose allelic variants might reduce β-lactam and carbapenem influx through altered membrane permeability (*5*,*13*). *C. coli* is less frequently associated with outbreaks, despite its broad ecologic niche, which includes broilers, pigs, and wild birds, emphasizing the risk for zoonotic transmission and environmental contamination (*1*,*14*,*15*).

Taking advantage of ongoing WGS-based surveillance in Portugal, we aimed to characterize the multidrug-resistant *C. coli* sequence type (ST) 10042 strain, which is associated with a peak of *C. coli* infections in Portugal and has been detected in other countries (Germany, Luxembourg, France, Ireland, Denmark, the Netherlands, the United Kingdom, and Spain) and diverse sectors (human, animal, food, and environment). The objectives were to elucidate its transmission dynamics and AMR determinants, assess its potential public health significance as an emerging *C. coli* lineage, and provide a more refined perspective on *Campylobacter* surveillance and the pathogen’s overall burden.

## Materials and Methods

### *Campylobacter* Surveillance in Portugal

Since 2013, the Portuguese National Reference Laboratory for Gastrointestinal Infections (NRL-GI) at the National Institute of Health Doutor Ricardo Jorge has coordinated sentinel laboratory-based surveillance of human campylobacteriosis, involving hospital laboratories from the 3 (of 5 total) most populated geographic areas in mainland Portugal (the North, Centre, and Metropolitan Lisbon Area regions) and from 2 private laboratory networks covering the entire mainland. The network routinely submits *Campylobacter* isolates on a monthly basis, covering the entire year (*16*). Since 2022, WGS has been systematically implemented, achieving ≈90% coverage for *C. coli* WGS and antimicrobial susceptibility testing (AST).

Animal and food-chain surveillance programs are conducted by the National Institute for Agricultural and Veterinary Research. Livestock isolates are recovered from cattle, broiler, and turkey cecal contents at slaughter under the Europe monitoring framework established by Commission Decision 2020/1729 (*17*). We obtained environmental isolates and additional isolates from animals and meat products from collaborative research projects, including screening in poultry meat in retail (2022**–**2025) (A. Nunes et al., unpub. data) and targeted studies on dogs (2022–2023) (M.-L. Lemos et al., unpub. data).

### *C. coli* ST10042 Study Sample

This study included *C. coli* ST10042 isolates identified since the start of WGS-based surveillance in Portugal in 2022. To ensure temporal completeness, we retrospectively sequenced all *C. coli* isolates received at the NRL-GI during 2016–2021 exhibiting the conserved ST10042 AMR phenotype. ST10042 had been previously underrecognized in public databases; WGS surveillance in Portugal revealed it as a predominant and relevant ST.

Collaborating partners in Germany, Luxembourg, France, Ireland, Denmark, and the Netherlands provided additional *C. coli* ST10042 isolates, generated through national WGS surveillance or research monitoring. Sampling, WGS coverage, and methodologies varied across countries and sectors (Table 1). The dataset was complemented with a limited number of genomes from the United Kingdom and Spain, retrieved from the PubMLST database (http://pubmlst.org), last accessed in January 2026. In total, we integrated 217 *C. coli* ST10042 isolates into the study (Appendix Table). 

**Table 1:** *Campylobacter* spp. whole-genome sequencing–based surveillance by country and source in study of multisectoral emergence of multidrug-resistant *Campylobacter coli* sequence type 10042 lineage, Europe, 2018–2025*

Country (sector)	*Campylobacter* WGS surveillance and coverage
Ireland (human)	WGS-based surveillance since February 2019. Approximately 10% of national *Campylobacter* cases sequenced annually; ≈10% of sequenced isolates are *C. coli*.
Luxembourg (human, animal, food)	WGS-based surveillance since 2013. 100% of isolates are sequenced.
Netherlands (human)	WGS-based surveillance since May 2021. ≈400–500 isolates selected annually; ≈90% of sequenced isolates are *C. jejuni* and ≈10% are *C. coli*.
Netherlands (animal, food)	No routine WGS. Sequencing performed on subsets since 2000. During 2021–2022 monitoring, 549 *C. jejuni* and 960 *C. coli* isolates were recovered, 540 were sequenced.
Germany (human)	WGS-based surveillance since 2019. 100% of isolates are sequenced.
France (human)	WGS-based surveillance since 2024. ≈50% of received *C. coli* are sequenced.
Portugal (human)	WGS-based surveillance since 2022. 90% of *C. coli* isolates are sequenced.
Portugal (animal, food)	No routine WGS. Sequencing performed on subsets.

*WGS, whole-genome sequencing.

### Antimicrobial Susceptibility Testing

As part of the surveillance system in Portugal, AST was performed for 4 priority antimicrobial drugs (ciprofloxacin, erythromycin, tetracycline, and gentamicin) by disk diffusion (Bio-Rad Laboratories, https://www.bio-rad.com), using the breakpoints established by European Committee on Antimicrobial Susceptibility Testing (*18*). AST was also performed for 3 optional antibiotics (ampicillin, amoxicillin/clavulanic acid, and ertapenem) by MIC determination using E-test strips (bioMérieux, https://www.biomerieux.com), using the interpretive criteria established by the Comité de l’antibiogramme de la Société Française de Microbiologie (*19*), as previously described (*16*).

### Whole-Genome Sequencing

For the *C. coli* ST10042 isolates from the NRL-GI in Portugal, we extracted total DNA using the Viral NA SV Kit on a MagNA Pure 96 System (Roche, https://www.roche.com) and quantified it using Qubit (Thermo Fisher Scientific, https://www.thermofisher.com), followed by Nextera XT library preparation (Illumina, https://www.illumina.com). We performed paired-end sequencing (2 × 250 bp or 2 × 150 bp) on MiSeq, NextSeq 550, or NextSeq 2000 platforms at National Institute of Health Doutor Ricardo Jorge, according to manufacturer instructions. Isolates from collaborators were provided as raw sequencing (Table 1).

All isolates underwent read quality assessment, species confirmation, and de novo assembly using the INNUca version 4.2.2 pipeline (https://github.com/B-UMMI/INNUca), as previously described (*5*). We annotated draft genome sequences with RAST server version 2.05 (*20*).

### Genomic Characterization of *Campylobacter coli* ST10042

We determined in silico MLST using mlst version 2.18.1 (https://github.com/tseemann/mlst) and performed *porA* typing using the PubMLST scheme based on Dingle et al. (*21*). We identified AMR-related genes and mutations by querying genome assemblies against ResFinder version 4.7.2 (https://github.com/genomicepidemiology/resfinder), CARD 3.3.0/RGI 6.0.3 (https://card.mcmaster.ca/analyze/rgi), and AMRFinderPlus version 4.0.3 (https://github.com/ncbi/amr). We defined positive matches using minimum thresholds of 70% coverage and 80% nucleotide identity. We identified additional mutations in promoter regions of the *cmeRABC* efflux pump operon and *bla*_OXA-61_, assemblies by mapping assemblies against CIT382 (GenBank accession no. AY598796.4) and CCUG11283 (VZOM01000001.1) strains using Snippy version 4.6.0 (https://github.com/tseemann/snippy) with default parameters.

### Comparative Genomic Analysis

We performed a comparative genomic analysis for the complete *C. coli* ST10042 dataset (N = 217) and the subset of isolates from Portugal (n = 104) using the PubMLST *Campylobacter* core-genome MLST version 1 scheme (1,343 loci) (*22*). We conducted allele calling with chewBBACA version 3.1.2 (https://github.com/B-UMMI/chewBBACA) using default parameters (bsr_theshold: 0.6, size_threshold: 0.2). Only exact and inferred allele assignments were retained for the construct of allelic profile matrices; we treated other allelic classification categories (https://chewbbaca.readthedocs.io/en/latest/index.html) as missing loci.

We performed genetic clustering and epidemiologic associations (country, year, source, AMR profile) using ReporTree version 2.5.4 (https://github.com/insapathogenomics/ReporTree) (*23*). We generated minimum-spanning trees with the MSTreeV2 algorithm implemented in GrapeTree. We excluded assemblies with <95% loci called in the core-genome MLST scheme. We defined clusters using a maximum threshold of 5 allelic distances (ADs).

## Results

### Emergence and Temporal Dynamics of *C. coli* ST10042 in Portugal

Under the scope of campylobacteriosis surveillance, an increase in *C. coli* infections was observed in 2022; *C. coli* infections accounted for 19% (83/441) of isolates processed at the NRL-GI in Portugal, compared with 14% (47/334) in 2021. *C. coli* ST10042 was predominant in Portugal in 2022, representing 30% (25/83) of *C. coli* cases, detected in the metropolitan Lisbon area (44% [11/25]), the northern (36% [9/25]) and central (20% [5/25]) regions. Circulating at much lower frequencies that year, ST832 (10% [8/83]) was most common, followed by ST827 and ST1595 (6% each [5/83]), and ST825, ST855, ST3017, and ST5659 (5% each [4/83]). All those STs belong to ST828 clonal complex.

After a 2022 peak, ST10042 declined to 14% (11/79) in 2023 and stayed at 15% (14/95) in 2024, before rising to 24% (29/120) of *C. coli* infections in 2025. Retrospectively, it was lower in preceding years: 15% (7/47) in 2021 and 1 case in September 2020 (1/42). Overall, ST10042 was Portugal's most frequent *C. coli* ST during 2021–2025 and its 2022 and 2025 prevalence coincided with and drove overall infection peaks (Figure 1). Consistent with previous data (*16*), cases lacked a clear seasonal pattern and occurred year-round.

**Figure 1:**
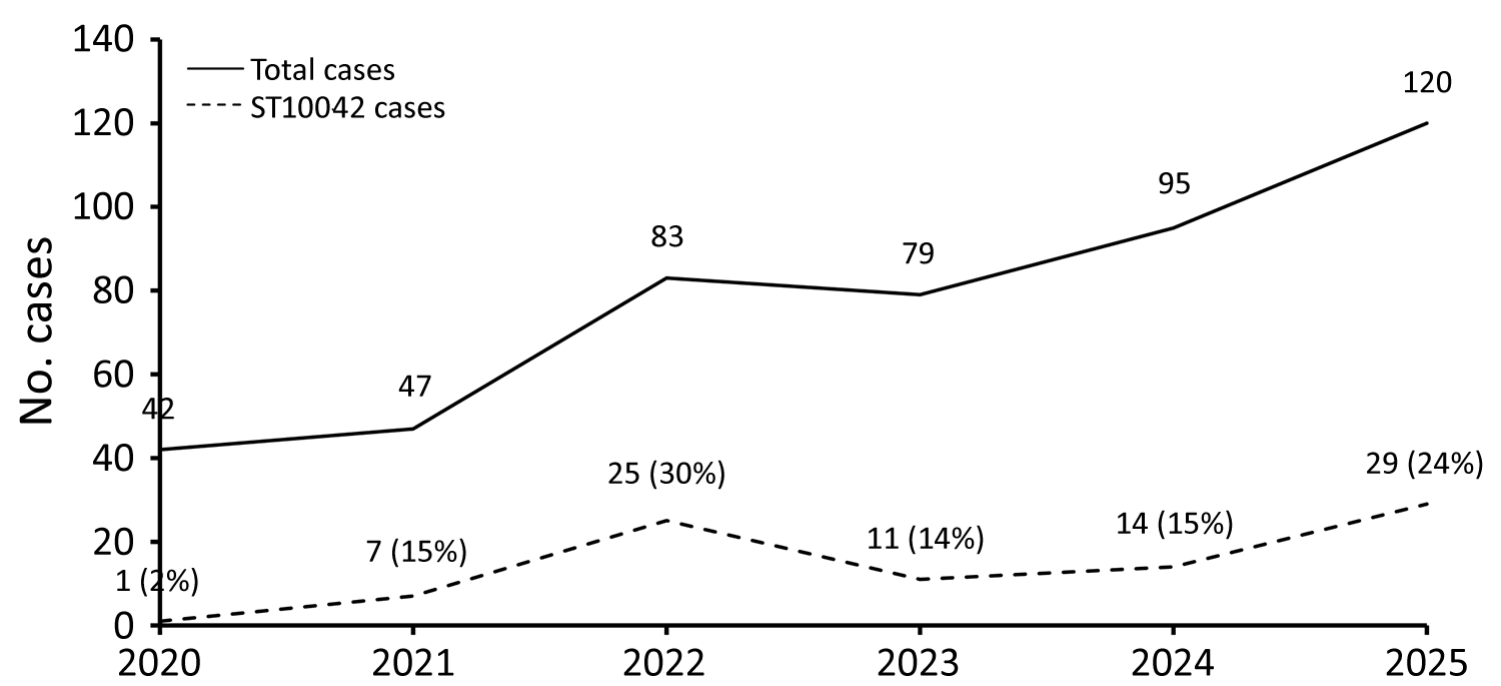
Temporal trend of *Campylobacter coli* human isolates (n = 466) and ST10042 isolates (n = 87), Portugal, 2020–2025, in study of multisectoral emergence of multidrug-resistant *C. coli* ST10042 lineage, Europe, 2018–2025. ST, sequence type.

### Epidemiology of *C. coli* ST10042 in Europe

Given the increase in *C. coli* infections in Portugal in 2022 driven by the emergence of ST10042, we conducted an extended investigation of Europe. In total, we included 217 *C. coli* ST10042 isolates collected during August 2018–December 2025 in the study. The isolates originated from 9 countries in Europe, and most were human isolates (n = 184 cases); of those, Portugal contributed the largest number (n = 85), followed by Germany (n = 30), Luxembourg (n = 21), France (n = 12), Ireland (n = 11), Denmark (n = 9), the Netherlands (n = 8), the United Kingdom (n = 7), and Spain (n = 1). Portugal, Luxembourg, the Netherlands, and Ireland also contributed animal/food isolates (n = 30); environmental isolates were detected only in Portugal and Luxembourg (n = 3) (Table 2). Within the study sample, the earliest detections (2018–2019) were reported in Luxembourg, Germany, and Ireland (Table 2; Figure 2). During 2020–2025, other countries reported ST10042 sporadically and at low frequency, whereas Portugal experienced a marked increase in 2022 and 2025, corresponding to the highest annual totals of reported cases during the overall period (Table 2; Figure 2).

**Table 2:** Distribution of isolates (N = 217) by year, country and source in study of multisectoral emergence of multidrug-resistant *Campylobacter coli* sequence type 10042 lineage, Europe, 2018–2025*

Year of isolation	Country of isolation	No. isolates by source				Total no. isolates
		Human	Animal	Food	Environmental	
2018	Luxembourg	1	0	0	0	1
2019	Germany	1	0	0	0	1
	Luxembourg	1	1	1	0	3
	Ireland	1	0	0	0	1
2020	Portugal	1	0	0	1	2
	Germany	9	0	0	0	9
	Luxembourg	3	0	3	0	6
	France	2	0	0	0	2
	Denmark	3	0	0	0	3
2021	Portugal	5	1	1	0	7
	Germany	7	0	0	0	7
	Luxembourg	3	0	0	1 (*24*)	4
	France	1	0	0	0	1
	Ireland	1	0	0	0	1
	Denmark	2	0	0	0	2
	The Netherlands	0	3	0	0	3
2022	Portugal	25	4	0	1	30
	Germany	2	0	0	0	2
	Luxembourg	4	1	1	0	6
	France	1	0	0	0	1
	Ireland	8	1	0	0	9
	The Netherlands	1	1	0	0	2
	United Kingdom	3	–	–	–	3
2023	Portugal	11	1	4	0	16
	Germany	6	0	0	0	6
	Luxembourg	2	0	0	0	2
	France	1	0	0	0	1
	Denmark	3	0	0	0	3
	The Netherlands	2	0	0	0	2
	United Kingdom	4	–	–	–	4
	Spain	1	–	–	–	1
2024	Portugal	14	0	6	0	20
	Germany	3	0	0	0	3
	Luxembourg	4	0	0	0	4
	France	3	0	0	0	3
	Ireland	1	1	0	0	2
	The Netherlands	4	0	0	0	4
2025	Portugal	29	0	0	0	29
	Germany	2	0	0	0	2
	Luxembourg	3	0	0	0	3
	France	4	0	0	0	4
	Denmark	1	0	0	0	1
	The Netherlands	1	0	0	0	1
Total		184	14	16	3	217

*Dash indicates no available data because correspondent countries’ data were retrieved from PubMLST.

**Figure 2:**
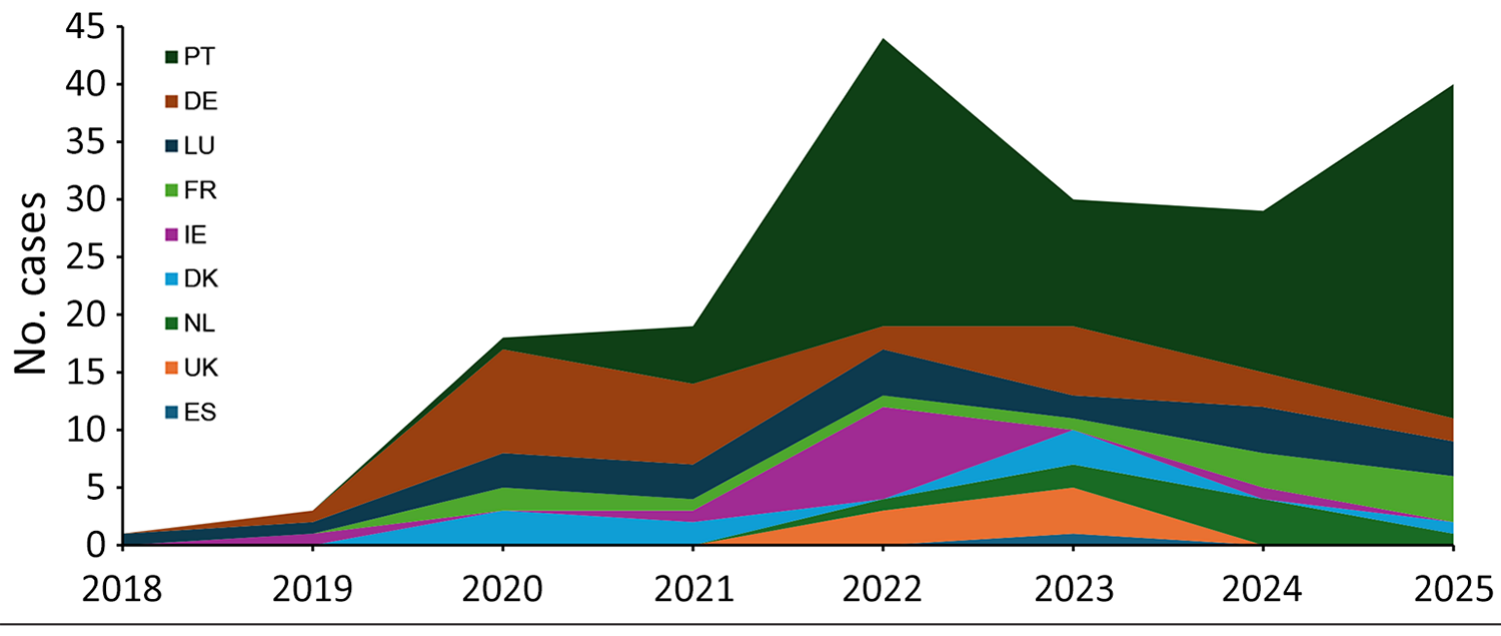
Annual distribution of *Campylobacter coli* ST10042 human isolates (n = 184) by country in study of multisectoral emergence of multidrug-resistant *C. coli* ST10042 lineage, Europe, 2018–2025. DE, Germany; DK, Denmark; ES, Spain; FR, France; IE, Ireland; LU, Luxembourg; NL, the Netherlands; PT, Portugal; ST, sequence type; UK, United Kingdom.

### Animal, Food, and Environmental Isolates

Food-derived isolates (n = 16) and animal-derived isolates (n = 14) collected during 2019–2024 were identified from Portugal (n = 17), Luxembourg (n = 7), the Netherlands (n = 4), and Ireland (n = 2); of those, 83% (25/30) originated from poultry. The food isolates included 11 recovered from raw poultry meat in Portugal (chicken [n = 9] and turkey [n = 2]) in 2021, 2023 and 2024 and 5 recovered from chicken-based prepared dishes in Luxembourg in 2019, 2020, and 2022. A total of 13 animal isolates (93% [13/14]) originated from livestock; those consisted of 5 from Portugal (chicken [n = 2], turkey [n = 2], and cattle [n = 1] in 2021–2023), 4 from the Netherlands (poultry [n = 2], cattle [n = 2] in 2021–2022), 2 from Luxembourg (poultry [n = 1] and cattle [n = 1] in 2019 and 2022) and 2 from Ireland (poultry [n = 2] in 2022 and 2024). One isolate (7% [1/13]) was recovered from a dog in Portugal (2023). In addition, 3 environmental isolates were recovered from surface waters, 2 from Portugal (2020 and 2022) and 1 from Luxembourg (2021).

### AMR Phenotype and Genotype of *C. coli* ST10042

We performed AST on the *C. coli* isolates from the NRL-GI in Portugal (85 human, 10 food, 2 environmental, and 1 companion animal). All were resistant to ciprofloxacin, tetracycline, and ampicillin (MIC >256 mg/L) (Table 3). All but 2 isolates were also resistant to amoxicillin/clavulanic acid (MIC = 12–64 mg/L); 1 isolate exhibited a higher MIC (>256 mg/L). Regarding ertapenem, all but 2 isolates exhibited increased MIC values (MIC = 0.38–1.0 mg/L) compared with those typically observed among susceptible *C. coli* isolates (MIC = 0.016–0.125 mg/L) according to previously published data (*16*), which we defined as decreased susceptibility (Table 3). The 2 exceptions had lower MICs for amoxicillin/clavulanic acid and ertapenem: 8 and 0.125 mg/L for PT-85 and 0.094 and 0.032 mg/L for PT-93 (Table 3). We detected resistance to erythromycin (MIC >256 mg/L) in 2 isolates (PT-7 and PT-28) (Table 3).

**Table 3:** *Campylobacter coli* ST10042 isolates phenotypic and genotypic (antimicrobial resistance profiles in study of multisectoral emergence of multidrug-resistant *C. coli* ST10042 lineage, Europe, 2018–2025*

Class	Antibiotic	Phenotype, n = 98, NRL-GI Portugal isolates				Genotype, N = 217, all isolates	
		Disk, mm, or MIC, mg/L	Susceptibility	No. tested/ no. positive		Resistance determinants	No. tested/ no. positive
Fluoroquinolones	Ciprofloxacin	6 mm	R	98/98		GyrA Thr86Ile	217/217
Tetracyclines	Tetracycline	6 mm	R	98/98		*tet*(32/O/32)	213/217
						*tet*(O)	4/217
β-lactams	Ampicillin	>256 mg/L	R	98/98		*bla*_OXA-61_	216/217
						*bla*_OXA-61_ promoter: −57G>T	
	Amoxicillin/clavulanic acid	>256 mg/L	R	1/98		*bla*_OXA-61_	1/217
						*bla*_OXA-61_ promoter: −57G>T	
						−69delA	
						*porA* 35	
		12–64 mg/L	R	95/98		*bla*_OXA-61_	211/217
						*bla*_OXA-61_ promoter: −57G>T	
						*porA* 35/1006/4736	
		8/0.094 mg/L	S	2/98		*bla*_OXA-61_	4/217
						*bla*_OXA-61_ promoter: −57G>T	
		–				*bla*_OXA-61_	1/217
						*porA* 35	
Carbapenems	Ertapenem	0.30–1.0 mg/L	DS	96/98		*porA* 35/1006/4736†	213/217
		0.032/0.125 mg/L	S	2/98		x	4/217
Macrolides	Erythromycin	6 mm/>256 mg/L	R	2/98		*23S rRNA* (A2075G)	6/217
						*23S rRNA* (A2074G)	1/217
						*23S rRNA* (A2074C)	1/217
		>24 mm	S	96/98		x	209/217

*Dash indicates no antimicrobial susceptibility testing was performed. X indicates absence of resistance determinants. DS, decreased susceptibility; NRL-GI, National Reference Laboratory for Gastrointestinal Infections; R, resistant; S, susceptible; ST, sequence type. †*porA* 1006 and *porA* 4736 differ from allele 35 by a single synonymous substitution. Other genetic determinants included: *aadE* (n = 1/217), *aad9* (n = 1/217), *aph(3′)-IIIa* (n = 1/217) and *ant (*6*)-Ia* (n = 3/217). Gentamicin was tested and found to be susceptible for NRL-GI Portuguese isolates (>17 mm). Zone diameter breakpoints for ciprofloxacin (S >26 mm), tetracycline (S >30), and erythromycin (S >24 mm), and MIC breakpoint for erythromycin (S <8 mg/L) established by EUCAST v16.1 were used; interpretive criteria for ampicillin (S <8 mg/L), amoxicillin/clavulanic acid (S <8 mg/L), and ertapenem (S <1 mg/L), established by the CA-SFM V.1.1 were used.

In silico analysis of the 217 *C. coli* ST10042 isolates revealed an overall conserved resistance profile, harboring known genetic determinants of resistance to ciprofloxacin (GyrA Thr86Ile), tetracycline [*tet*(O/32/O)/*tet*(O)], and ampicillin (*bla*_OXA-61_ promoter: −57G>T) (Table 3). The single isolate from Portugal exhibiting MIC >256 mg/L for amoxicillin/clavulanic acid (PT-86) harbored an additional deletion (−69delA) in the *bla*_OXA-61_ promoter (Table 3). We identified erythromycin resistance–associated mutations in *23S rRNA* in 8 isolates (Table 3; Appendix Table). All but 6 isolates carried the *porA* allele 35 (Table 3; Appendix Table). Of note, the 2 Portugal isolates with lower MICs to amoxicillin/clavulanic acid and ertapenem harbored the *porA* allele 1512 instead. All isolates carried the *cmeRABC* efflux pump operon, for which no previously described resistance-associated point mutations or the resistance-enhancing *cmeB* variant (GenBank accession no. KT778508.1) were identified (*25*,*26*).

### Genetic Diversity of *C. coli* ST10042

Cluster analysis of the 217 *C. coli* ST10042 isolates assessed genomic diversity and relatedness. We generated 2 minimum-spanning trees, 1 including all 104 Portugal isolates (Figure 3) and another including 211 filtered isolates from the full study sample (Figure 4). The 104 Portugul isolates (2020–2025) exhibited high genetic diversity, and consisted of 1 main cluster of 28 isolates, 15 smaller clusters of 2–4 isolates (totalizing 35 isolates), and 41 singletons (Figure 3).

**Figure 3:**
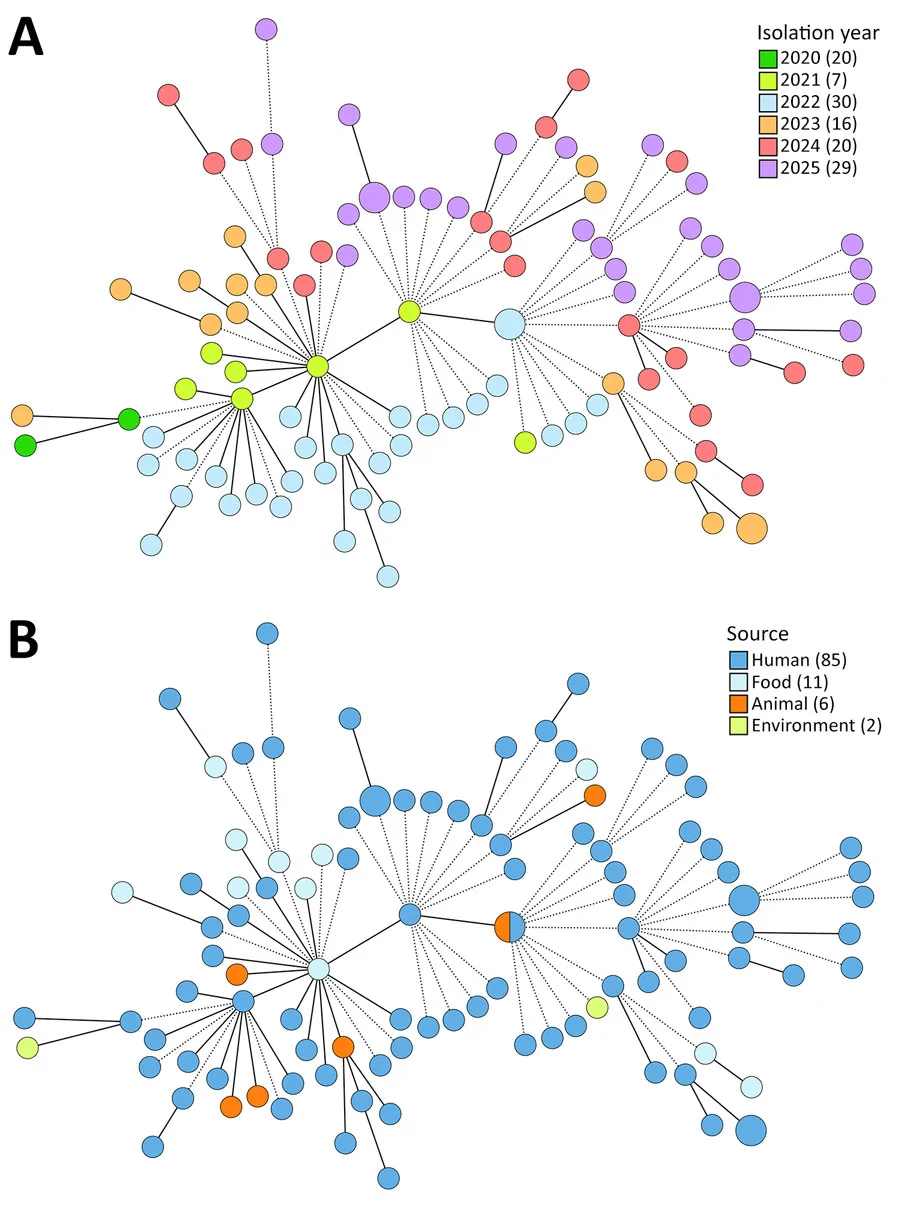
Diversity of the *Campylobacter coli* sequence type 10042 isolates from Portugal in study of multisectoral emergence of multidrug-resistant *C. coli* sequence type 10042 lineage, Europe, 2018–2025. Minimum-spanning tree of 104 Portugal isolates shows genetic diversity assessed using a gene-by-gene approach based on the PubMLST core-genome MLST v1 scheme (1,343 loci). Minimum-spanning tree was generated using the MSTreeV2 algorithm in GrapeTree (https://github.com/achtman-lab/grapetree) and based on allelic differences. Filled circles (sizes proportional to the number of isolates) represent unique allelic profiles. Nodes are colored by year of isolation (A) and source (B). Solid lines indicate genetic clusters defined by an allelic distance <5, whereas dashed lines indicate an allelic distance >5.

**Figure 4:**
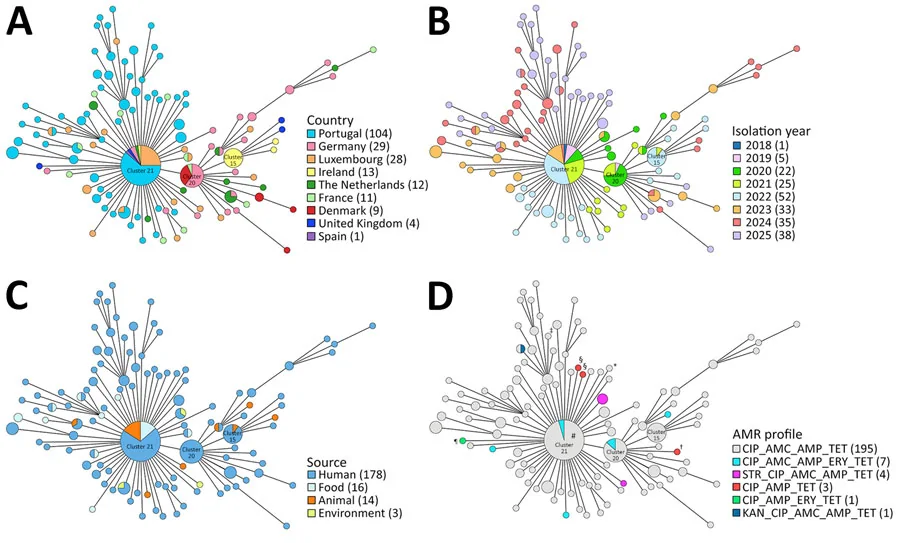
Diversity of *Campylobacter coli* sequence type 10042 isolates in study of multisectoral emergence of multidrug-resistant *C. coli* sequence type 10042 lineage, Europe, 2018–2025. Minimum-spanning tree of 211 isolates from 9 countries in Europe (Portugal, Germany, Luxembourg, France, Ireland, Denmark, the Netherlands, the United Kingdom, and Spain). Genetic diversity was assessed using a gene-by-gene approach based on the PubMLST cgMLST v1 scheme (1,343 *loci*). Minimum-spanning tree was generated using the MSTreeV2 algorithm in GrapeTree and based on allelic differences. Filled circles (sizes proportional to the number of isolates) indicate unique allelic profiles. Nodes are collapsed at an allelic distance <5 and colored according to country (A), year (B), source (C), and antimicrobial resistance profile (D). For clarity, only the 3 main clusters (*15*,*20*,*21*) are labeled. In panel D, all isolates harbor *bla*_OXA-61_ and the −57G>T promoter mutation. Isolates marked with a symbol additionally carry the promoter deletion or a different *porA*. *bla_OXA-61_ + promoter(–57G>T; *delA*-69)_*porA*(35) (n = 1); †*bla*_OXA-61_  + promoter(–57G>T)_*porA*(4736) (n = 1); ‡*bla*_OXA-61_ + promoter(–57G>T)_*porA*(914) (n = 1); §*bla*_OXA-61_ + promoter(–57G>T)_ *porA*(1512) (n = 2); ¶*bla*_OXA-61_ + promoter(–57G>T)_ *porA*(4735) (n = 1); #*bla*_OXA-61_ + promoter(–57G>T)_*porA*(1006) (n = 1).

Among the 85 human isolates, 57% (4/7) from 2021 and 43% (13/30) from 2022 grouped in the main cluster (Figure 3, panel A). Conversely, from 2023 onward, the population structure became more dispersed and genetically heterogeneous; human isolates primarily formed small clusters or singletons (81% in 2023, 95% in 2024, and 100% in 2025) (Figure 3, panel A). The main cluster consisted of 20 human isolates (2021–2024), mostly from 2021–2022 (85% [17/20]), and 8 nonhuman isolates: 5 livestock (chicken, turkey, or cattle collected in 2021–2022) and 3 poultry meat isolates from 2021, 2023, and 2024 (Figure 3, panels A, B). We observed no clear geographic aggregation.

Considering the complete dataset of 211 isolates, we observed a high level of genetic diversity. We identified a total of 28 clusters (defined as >2 isolates) that accounted for 129 isolates, whereas 82 isolates were singletons. Three clusters were predominant: cluster 21 (44 isolates), cluster 20 (15 isolates), and cluster 15 (10 isolates) (Figure 4).

Cluster 21 (Figure 4) was the largest cluster, embedding the main Portugal cluster (Figure 3) within a broader lineage within Europe. The human isolates were predominantly from Portugal (n = 19 [2021–2023]) and Luxembourg (n = 7 [2018–2022]), with sporadic isolates from Germany (n = 2 [2021]), Ireland (n = 1 [2019]), the United Kingdom (n = 1 [2023]), and Spain (n = 1 [2023]) (Figure 4, panels A, B). That cluster also included 13 source isolates: 7 livestock from Portugal (chicken, n = 2; turkey, n = 2; cattle, n = 1 [2021–2022]), Luxembourg (cattle, n = 1 [2019]), and the Netherlands (cattle, n = 1 [2021]), and 6 food samples from Portugal (poultry meat, n = 3 [2021, 2023, and 2024]) and Luxembourg (chicken-based dishes, n = 3 [2019–2020]) (Figure 4, panel C). The second largest cluster, cluster 20, consisted of 15 human isolates from Germany (n = 9 [2019–2021]), Denmark (n = 5 [2020–2021]), and France (n = 1 [2020]); the isolate from France came from a patient residing in Strasbourg, which borders Germany (Figure 4, panels A–C). Finally, cluster 15 consisted of 9 human isolates and 1 chicken isolate, all from Ireland and collected during 2021–2022 (Figure 4, panels A–C).

Overall, both minimum-spanning trees (Figure 3, panel A; Figure 4, panel B) revealed increasing genetic diversification over time; isolates progressively dispersed toward the periphery as singletons or smaller clusters. Although the core multidrug-resistance profile remained conserved across the sample, specific AMR phenotypes and genotypes, including the *porA* allele variants and the −69A deletion in *bla*_OXA-61_ promoter (associated with high-level resistance to amoxicillin-clavulanic), occurred mainly among peripheral isolates (Figure 4, panel D).

## Discussion

Since *Campylobacter* WGS surveillance was implemented in Portugal, key achievements have been reached, such as the detection of relevant STs, transmission events, AMR profiles, genomic clusters, and attribution of sources (*16*,*27*). Leveraging the high resolution of this surveillance, we identified previously unrecognized clusters of an emerging ST and assembled a study sample consisting of 217 *C. coli* ST10042, from human (n = 184) and nonhuman (animal, food, and environmental) (n = 33) isolates, encompassing 9 countries in Europe over 7 years (2018–2025).

ST10042, belonging to the globally disseminated ST828 clonal complex, first drew attention in Portugal in 2022, in which it accounted for 30% of human *C. coli* infections and demonstrated consistency with a poultry-related outbreak. Despite low international reports, collaboration revealed its presence across databases from collaborators across Europe, forming distinct genetic clusters across countries and sectors. The detection of ST10042 in poultry, cattle, dog, surface water, and human sources reinforces its ecologic plasticity and adaptability, underscoring the challenge of implementing control measures across diverse sources (*28*). Cluster 21, the largest cluster, consisted mainly of isolates from Portugal and Luxembourg, along with sporadic cases from Germany, Ireland, the United Kingdom, and Spain. Other larger clusters included cluster 15 (Ireland) and cluster 20 (Germany–Denmark). Although *C. coli* is typically associated with sporadic, unrelated infections (*7*), our WGS-based surveillance we demonstrated that major *C. coli* lineages form genetically related clusters with transnational and cross-sectoral dissemination, challenging previous population structure assumptions. Of note, cross-country cluster detection and comparison depend on national surveillance variations, such as sampling strategies and sequencing coverage. Although all 6 participating nations have longstanding public health WGS surveillance, coverage spans from 10% in Ireland to 100% in Germany (Table 1), differences that likely bias cluster resolution, sensitivity, and the apparent geographic distribution of ST10042.

In Portugal, *C. coli* ST10042 emerged in September 2020 and persisted through 2025, peaking in 2022 and 2025 (Figure 1). Most 2021–2022 isolates belonged to a large genetic cluster (Figure 3) integrated within cluster 21 from Europe, whereas 2023–2025 isolates formed smaller clusters or singletons (Figure 3, panel A), which was also observed at the Europe-wide level (Figure 4, panel B). Those patterns support ongoing circulation and diversification of ST10042 lineage, highlighting its outbreak potential, without excluding unsampled reservoirs.

Cluster 21 (2018–2024) (Figure 4, panel C) evidences interconnected transmission across animal, food, and human compartments, potentially through food production or animal trade. The predominance of poultry-derived isolates within the cluster (77% [10/13]), together with the fact that 83% (25/30) of animal and food-related cases in the overall study were traced to poultry, underscores the central role of this reservoir in *C. coli* transmission and the importance of strengthened surveillance and targeted control measures in the poultry production chain (*2*,*9*,*12*,*28*). The early detection of isolates from Luxembourg (2018–2020) might reflect differences in surveillance coverage and sampling intensity rather than a primary point of introduction. At the same time, the presence of a substantial Portuguese community in Luxembourg (on average 15% of residents) (https://statistiques.public.lu/en.html) that contributes to the availability of Portuguese food products through import and retail networks potentially provides a cross-border contamination pathway. However, without detailed epidemiologic and supply-chain data, that possibility remains speculative (*23*,*27*).

Cluster 15 from Ireland, which clustered chicken and human isolates and showed temporal concentration (2021–2022) (Figure 4, panels A and B), was consistent with an outbreak, possibly linked to chicken-born contamination in the food-chain. The Germany–Denmark cluster 20 emphasized the cross-broader circulation of ST10042 lineage (Figure 4, panel A), which was potentially sustained by an unsampled contamination source, such as a food item.

Overall, *C. coli* ST10042 lineage exhibits stable multidrug resistance, including decreased ertapenem susceptibility; genotype-phenotype concordance is strong. It exhibited resistance to both ciprofloxacin and tetracycline, consistent with the widespread occurrence of resistance to these antibiotics across the EU (*29*). In contrast, macrolide resistance was uncommon in this lineage, in agreement with the overall situation in Europe, where erythromycin resistance in *C. coli* remains substantially lower than for ciprofloxacin and tetracycline, although moderate levels are still reported in some countries, including Portugal and Spain (*29*). Resistance to amoxicillin/clavulanic acid and carbapenems remains rare (*5*,*30*); therefore, ST10042 stands out from other *C. coli* populations, raising concern given the critical role of β-lactams and carbapenems in treating invasive or complicated *Campylobacter* infections (*5*,*31*,*32*). That concern is amplified by ST10042 becoming the predominant *C. coli* ST in Portugal (2021–2025) and by its capacity for sustained, transnational, cross-sectoral circulation and dissemination. Those factors might reflect a well-established, stable lineage that confers selective advantage under antimicrobial pressure from human medicine and food or animal production systems (*33*), highlighting the need for strengthened antimicrobial stewardship.

Resistance to amoxicillin/clavulanic acid had only been previously linked to the −69A deletion in *bla*_OXA-61_ promoter (*30*). Although most *C. coli* ST10042 isolates in Portugal were resistant, only 1 carried that deletion, conferring high-level resistance (MIC >256 mg/L). Our data suggest that *porA* allele 35 influences susceptibility to amoxicillin/clavulanic acid and ertapenem. The cumulative presence of determinants, specifically the *porA* 35 allele, the –57 G→T transversion, and the −69A deletion in the *bla*_OXA-61_ promoter, appears to contribute to elevated amoxicillin/clavulanic acid resistance, whereas isolates lacking the deletion show lower resistance MICs.

The main limitations of this study relate to both epidemiologic and surveillance-related constraints. The lack of detailed epidemiologic metadata precludes confirmation of transmission routes or outbreak causality. Outbreak definition also requires validated genomic cutoffs. Overrepresentation of Portugal and differences in WGS surveillance coverage between countries resulted in sampling bias. Finally, a deeper understanding of the link between AMR genotypes and observed phenotypes is also needed. Regarding ertapenem, the absence of established clinical breakpoints and corresponding clinical outcome data jeopardizes definitive interpretation of decreased susceptibility and its clinical and public health implications. Further studies correlating MIC distributions with clinical outcomes are needed to clarify those associations.

This study highlights coordinated One Health WGS surveillance for detecting epidemiologically relevant *C. coli* lineages and monitoring AMR across human, animal, food, and environmental sectors. Multicountry genomic data revealed prolonged, transnational, and multisectoral circulation of the multidrug-resistant *C. coli* ST10042 lineage driven by poultry. Because single-sector surveillance is insufficient to capture transmission dynamics, those findings emphasize the need for harmonized, standardized WGS surveillance frameworks across Europe, including consistent sampling strategies, harmonized analytical pipelines, and shared genomic databases to enable real-time cross-border comparison of high-risk clones. Future work should integrate longitudinal WGS with detailed epidemiologic metadata and systematic production-chain sampling, while advancing higher-resolution species-specific typing schemes. Our findings support EU-level One Health WGS surveillance aligned with the recent Commission Implementing Regulation (EU) 2025/179, which mandates cross-sector genomic surveillance of key foodborne pathogens, including *C. coli* (https://data.europa.eu/eli/reg_impl/2025/179/oj). 

Appendix Additional information about multisectoral emergence of multidrug-resistant *Campylobacter coli* sequence type 10042 lineage, Europe, 2018–2025.
